# Prevalence of Pain Management Techniques Among Adults With Chronic Pain in the United States, 2019

**DOI:** 10.1001/jamanetworkopen.2021.46697

**Published:** 2022-02-07

**Authors:** Cornelius B. Groenewald, Caitlin B. Murray, Marco Battaglia, M. Simona Scaini, Patrick D. Quinn

**Affiliations:** 1 Center for Child Health, Behavior and Development, Seattle Children’s Research Institute, Seattle, Washington; 2 Department of Anesthesiology and Pain Medicine, University of Washington, Seattle; 3 Department of Psychiatry, the University of Toronto, Toronto, Ontario, Canada; 4 Child, Youth, and Emerging Adults Program, Centre for Addiction and Mental Health, Toronto, Ontario, Canada; 5 Child and Youth Lab, Sigmund Freud University, Milan, Italy; 6 Department of Applied Health Science, School of Public Health, Indiana University, Bloomington

## Abstract

This cross-sectional study estimates the prevalence of opioid and nonopioid pain management techniques used by US adults with chronic pain.

## Introduction

The Centers for Disease Control and Prevention (CDC) used data from the 2019 National Health Interview Survey (NHIS) to estimate that 50.2 million US adults experience chronic pain.^1^ A second CDC publication from NHIS found that 22.1% of these US adults with chronic pain used prescription opioids.^2^ However, the CDC did not determine whether opioids were used for acute vs chronic pain and did not explore other pain management techniques used. The present study provides baseline information on opioid and nonopioid pain management techniques used for chronic pain and serves as a benchmark for evaluating the outcome of health care policies aimed at reducing prescription opioid use.^3^

## Methods

The NHIS is the principal source of information on the health of the civilian, noninstitutionalized US population. Race and ethnicity were self-reported by participants and categorized by the NHIS. The Seattle Children’s Hospital institutional review board deemed this study exempt from review because it did not constitute human participants research as the NHIS contains no protected health information. All participants in the NHIS provided oral informed consent. We followed the Strengthening the Reporting of Observational Studies in Epidemiology (STROBE) reporting guideline for cross-sectional studies. The 2019 NHIS response rate was 61.1% with a final sample of 31 916 participants.

Questions on pain management techniques appeared for the first time in the 2019 NHIS. Adults with chronic pain were asked to report on their use of 11 pain management techniques during the past 3 months (Table 1), which were coded by the study authors into 6 categories: (1) opioids for chronic pain; (2) opioids for acute pain; (3) physical, occupational, or rehabilitative therapy; (4) psychological and psychotherapeutic therapies (eg, cognitive behavioral therapy); (5) complementary therapies (chiropractic, massage, yoga or tai chi, mediation); or (6) other (self-management, pain peer support group, and other methods for pain management). Participants were not specifically asked about nonopioid pharmacological pain treatments. Survey-weighted prevalence rates for each pain management technique were calculated for selected sociodemographic characteristics. Data were analyzed using Stata version 14.2 (StataCorp) from August to December 2021. Hypothesis testing was 2-sided, and *P* < .05 was considered statistically significant.

**Table 1.** Use of Opioid and Nonopioid Pain Management Techniques During the Past 3 Months Among Adults With Chronic Pain in the US

Pain management technique	Adults		Estimated population, millions
	No. in sample	% (95% CI)	
Opioid pain management			
Acute pain indication only	413	5.8 (5.2-6.5)	2.9
Chronic pain indication	1133	15.2 (14.2-16.3)	7.6
Nonopioid pain management			
Physical, occupational, or rehabilitative therapy for pain	1466	18.8 (17.8-19.9)	9.4
Talk therapies, such as cognitive behavioral therapy	268	3.8 (3.3-4.4)	1.9
Complementary therapies			
Spinal manipulation or other forms of chiropractic care	861	11.6 (10.7-12.6)	5.8
Yoga or Tai Chi	632	8.5 (7.8-9.4)	4.3
Massage for pain	1244	17.6 (16.5-18.7)	8.8
Meditation, guided imagery, or other relaxation techniques	1167	15.6 (14.6-16.7)	7.8
Other therapies			
A chronic pain self-management program or workshop	374	5.1 (4.5-5.8)	2.6
Chronic pain peer support group	119	1.8 (1.5-2.3)	0.9
Any other approaches	2874	39.1 (37.3-40.9)	19.6

## Results

Among 31 916 participants, 17 261 (64%) were female; 4152 (13%) were Hispanic, 3483 (10.9%) were non-Hispanic African American, and 21 915 (68.5%) were non-Hispanic White; 22 621 (70.9%) were 18 to 64 years of age, with 9295 (29.1%) at least 65 years of age. Pain management techniques used by adults with chronic pain in the US are presented in Table 1. Additionally, we estimated that 54.7% (95% CI, 53.1%-56.3%) of adults with chronic pain only used nonopioid pain management techniques, whereas 10.7% (95% CI, 9.9%-11.6%) used both opioids and nonopioid techniques, 4.4% (95% CI, 3.8%-5.1%) only used opioids for chronic pain management, and 30.2% (95% CI, 28.8%-31.7%) did not report any pain management techniques during the past 3 months (data on respondents reporting no pain management is not presented in tables). The most commonly used nonopioid pain management technique was complementary therapies, which was used by 35.4% (95% CI, 33.9%-36.9%) of adults with chronic pain, followed by physical, occupational or rehabilitative therapies (18.8% [95% CI, 17.8%-19.9%] of adults with chronic pain). Only 3.8% (95% CI, 3.3%-4.4%) of adults with chronic pain used psychological or psychotherapeutic interventions. Other techniques reported, included self-management programs (5.1% [95% CI, 4.5%-5.8%]) and chronic pain peer support groups (1.8% [95% CI, 1.5%-2.3%]). In addition, 39.1% (95% CI, 37.3%-40.9%) of adults with chronic pain reported using other pain approaches not specifically captured in the data set. Prescription opioid use for chronic pain was more common among older age groups (eg, for chronic pain among adults aged 45 to 64 years: 19.3% [95% CI, 17.6%-21.2%] vs adults aged 18 to 44 years: 8.4% [95% CI, 6.8%-10.3%]), female individuals (eg, for chronic pain among female individuals: 16.9% [95% CI, 15.5%-18.5%] vs male individuals: 13.1% [95% CI, 11.7%-14.7%]), insured individuals (eg, for chronic pain among those insured: 16.3% [95% CI, 15.2%-17.4%] vs uninsured: 5.8% [95% CI, 3.9%-8.4%]), those with high school education or lower (eg, for chronic pain among those with high school or less: 17.0% [95% CI, 15.4%-18.8%] vs more than high school: 13.8% [95% CI, 12.6%-15.1%]), and was less common among those with higher annual income (eg, for chronic pain among those from households that made at least $100 000 annually: 8.7% [95% CI, 7.2%-10.5%] vs less than $35 000 annually: 19.8% [95% CI, 18.1%-21.5%]) (Table 2). Adults using complementary and psychological or psychotherapeutic interventions were more likely than those not using these techniques to be younger, female, and have higher educational attainment. Adults using physical, occupational, or rehabilitative therapy were more likely than those not using these treatments to be older, female, have medical insurance, and be more highly educated.

**Table 2.** Past 3-Month Prevalence of Adults Using Opioids and Nonopioid Pain Management Techniques for Chronic Pain in the US by Sociodemographic Characteristics, 2019

Characteristics	Adults, weighted % (95% CI)					
	Opioid		Nonopioid			
	Acute pain	Chronic pain	Physical/occupational therapy	Talk therapy	Complementary therapies	Other
Age category, y						
18-44	6.6 (5.3-8.2)	8.4 (6.8-10.3)	15.6 (13.4-17.9)	6.3 (5.0-7.9)	44.4 (41.4-47.4)	42.5 (39.4-45.8)
45-64	5.4 (4.5-6.6)	19.3 (17.6-21.2)	18.3 (16.7-20.0)	3.6 (2.9-4.5)	36.4 (34.2-38.6)	44.8 (42.5-47.2)
≥65	5.6 (4.7-6.7)	15.5 (13.9-17.1)	22.2 (20.4-24.1)	1.9 (1.4-2.7)	26.6 (24.7-28.5)	40 (37.6-42.4)
Biological sex at birth						
Male	5.2 (4.4-6.2)	13.1 (11.7-14.7)	16.7 (15.1-18.4)	3.1 (2.4-3.9)	31.1 (29.0-33.2)	39 (36.8-41.2)
Female	6.2 (5.4-7.2)	16.9 (15.5-18.5)	20.6 (19.1-22.1)	4.5 (3.7-5.4)	38.9 (37.0-40.9)	45.7 (43.6-47.9)
Race and ethnicity						
Hispanic	4.5 (3.0-6.7)	14.1 (11.1-17.9)	17.5 (14.5-21.0)	4.5 (2.8-7.2)	34.1 (29.7-38.9)	37.3 (32.7-42.1)
Non-Hispanic, Black/African American	4.4 (3.0-6.6)	17.9 (15.0-21.3)	19.8 (16.6-23.5)	2.9 (1.9-4.4)	28.9 (25.0-33.2)	36.3 (32.0-40.9)
Non-Hispanic, White	6.3 (5.6-7.1)	15.1 (14.0-16.3)	18.9 (17.7-20.2)	3.6 (3.0-4.3)	36.2 (34.6-37.8)	44.1 (42.1-46.1)
Other^a^	3.8 (1.8-7.7)	13 (8.6-19.2)	18.5 (14.1-23.9)	7.7 (4.9-11.8)	40.3 (33.6-47.4)	46.8 (40.7-53.1)
Annual household income, $						
0-34 999	4.8 (4.0-5.9)	19.8 (18.1-21.5)	18.1 (16.4-19.9)	4.2 (3.4-5.2)	29.7 (27.8-31.7)	39.3 (36.9-41.8)
35 000-74 999	5.7 (4.6-7.0)	15.3 (13.4-17.4)	16.7 (15.0-18.6)	3.5 (2.7-4.5)	34.6 (32.1-37.2)	42.4 (39.9-45.0)
75 000-99 999	5.8 (4.0-8.4)	12.5 (9.7-16.0)	20.3 (16.9-24.2)	4.3 (2.8-6.5)	36.9 (32.8-41.3)	46 (41.5-50.5)
≥100 000	7.5 (6.0-9.3)	8.7 (7.2-10.5)	22.5 (20.1-25.1)	3.5 (2.4-5.1)	45.4 (42.1-48.8)	47 (43.7-50.3)
Insurance						
Insured	6 (5.3-6.7)	16.3 (15.2-17.4)	20 (18.8-21.1)	4 (3.4-4.6)	35.7 (34.2-37.2)	43.4 (41.6-45.2)
Uninsured	4.3 (2.7-6.6)	5.8 (3.9-8.4)	8.5 (6.2-11.5)	2.7 (1.4-5.0)	32.7 (28.2-37.5)	36 (31.2-41.1)
Education						
High school or less	4.9 (4.1-5.9)	17 (15.4-18.8)	14.7 (13.3-16.2)	2.3 (1.7-3.2)	26.4 (24.5-28.5)	36.7 (34.3-39.1)
More than high school	6.6 (5.7-7.6)	13.8 (12.6-15.1)	22.4 (21.0-23.9)	5.1 (4.4-6.0)	43.2 (41.4-45.1)	47.9 (45.8-50.0)

^a^ Other races and ethnicities included non-Hispanic Asian only; non-Hispanic American Indian/Alaska Native only; non-Hispanic American Indian/Alaska Native and any other race; other single and multiple race.

## Discussion

This study found that adults with chronic pain in the US use a variety of pain management techniques, including opioids. While effective for some, opioids prescribed for chronic pain management remain an important determinant of the national opioid crisis.^4^ Nonpharmacologic and nonopioid pharmacologic therapies are preferred treatments for chronic pain, and it is encouraging to note that most adults with chronic pain use a combination of various nonopioid modalities for treatment.^3^ However, only 3.8% of participants reported using psychological therapies. Psychological therapy, including cognitive behavioral therapy, is effective for improving chronic pain, and our study indicates that it is underused.^5^ Limitations of this study include that information is subject to recall and social desirability bias. Moreover, 39.1% of adults with chronic pain reported using other pain approaches not specifically captured in the data set. Strengths of this study include the ability to examine opioid and nonopioid use for chronic pain captured in NHIS for the first time.

This cross-sectional study calculated national estimates of opioid and nonopioid pain management techniques used by adults with chronic pain. Pain management techniques vary by sociodemographic characteristics. Improved understanding of effective nonopioid pain management techniques is needed to reduce the reliance on opioids for chronic pain.

## References

[1] Zelaya CE, Dahlhamer JM, Lucas JW, Connor EM. Chronic pain and high-impact chronic pain among U.S. adults, 2019. NCHS Data Brief. 2020;(390):1-8. 33151145

[2] Dahlhamer JM, Connor EM, Bose J, Lucas JL, Zelaya CE. Prescription opioid use among adults with chronic pain: United States, 2019. Natl Health Stat Report. 2021;(162):1-9. doi:10.15620/cdc:107641 34524076

[3] Dowell D, Haegerich TM, Chou R. CDC guideline for prescribing opioids for chronic pain—United States, 2016. JAMA. 2016;315(15):1624-1645. doi:10.1001/jama.2016.1464 26977696 PMC6390846

[4] Volkow ND, Baler R. A prescription for better opioid prescribing? Nat Med. 2018;24(10):1496-1498. doi:10.1038/s41591-018-0214-4 30297898

[5] Williams ACC, Fisher E, Hearn L, Eccleston C. Psychological therapies for the management of chronic pain (excluding headache) in adults. Cochrane Database Syst Rev. 2020;8:CD007407. doi:10.1002/14651858.CD007407.pub4 32794606 PMC7437545

