# Error in Byline

**DOI:** 10.1001/jamanetworkopen.2022.8840

**Published:** 2022-04-06

In the Research Letter titled “Prevalence of Pain Management Techniques Among Adults With Chronic Pain in the United States, 2019,”^1^ published February 7, 2022, a coauthor’s name was misspelled. It should have appeared as Simona Scaini, PhD. This article has been corrected.^1^
